# Clinical characteristics of *Mycoplasma pneumoniae* pneumonia in children and construction of a severe case prediction model: a retrospective study from Yan’an, China

**DOI:** 10.3389/fped.2026.1789585

**Published:** 2026-03-25

**Authors:** Qiaohui Zha, Huirong Li, Yuxuan Li, Hairui Gao, Yuanxia Li, Kunpeng Jia

**Affiliations:** 1 Department of Pediatrics, Yanan University Affiliated Hospital, Yan’an, Shaanxi, China; 2 Department of Pediatrics, Yan’an People’s Hospital, Yan’an, Shaanxi, China

**Keywords:** children, clinical features, critical, mycoplasma pneumoniae, predictive model

## Abstract

**Objective:**

This study aims to systematically analyze the clinical characteristics of *mycoplasma pneumoniae* pneumonia (MPP) in community-acquired pneumonia in children, identify the risk factors for severe *mycoplasma pneumoniae* pneumonia (SMPP), construct a visual nomogram prediction model, and provide a theoretical basis for the early clinical identification and timely diagnosis and treatment of SMPP.

**Methods:**

A retrospective analysis was conducted using clinical data from 500 children diagnosed with MPP who had been admitted to Affiliated Hospital of Yan'an University and Yan'an People's Hospital from January 2023 to December 2024.The patients were divided into mild and severe groups. Statistical analyses involved group comparisons using appropriate tests for continuous and categorical variables, multivariate logistic regression to identify independent risk factors, and receiver operating characteristic (ROC) curves to evaluate predictive performance (*P* < 0.05 considered significant).

**Results:**

Univariate and multivariate analyses showed that elevated Erythrocyte Sedimentation Rate (ESR), elevated Lactate Dehydrogenase (LDH), lung consolidation and elevated D-dimer were independent risk factors for SMPP, while elevated Albumin(ALB) was an independent protective factor for SMPP (all *P* < 0.05). ROC curve analysis showed that the efficacy of each indicator in diagnosing SMPP from high to low was: ESR >ALB >LDH >lung consolidation >D-dimer. The area under the ROC curve (AUC) of the nomogram prediction model for SMPP based on the above risk factors was 0.963 (95% confidence interval CI: 0.949–0.978); Both internal and external validated calibration curve and decision curve analysis (DCA) results confirmed that the nomogram model had good predictive power and clinical applicability.

**Conclusion:**

The nomogram, with its intuitive graphical interface, can transform complex mathematical predictive models into convenient and accessible tools in clinical practice, and has significant clinical predictive value. Clinicians only need to add up the corresponding scores of the patient's indicators (LDH ≈ 22.5 points, ESR ≈ 40.5 points, albumin ≈ 57.5 points, D-dimer ≈ 23.5 points, lung consolidation ≈ 22 points, total ≈ 22.5 + 40.5 + 57.5 + 23.5 + 22 = 166 points), it can quickly assess the risk of developing SMPP [P (SMPP) ≈ 80%–90%], and this tool is particularly applicable in primary care scenarios.

## 1 Introduction

*Mycoplasma pneumoniae* (MP) is one of the important pathogenic microorganisms causing community-acquired pneumonia (CAP) in children. The *Mycoplasma pneumoniae* pneumonia (MPP) it causes accounts for 10%–40% of CAP in children (1). MP infection can occur throughout the four seasons. In northern China, it is more prevalent in autumn and winter, while in southern China, it is more common in summer and autumn. There is an epidemic cycle every 3–7 years, usually lasting 1–2 years, with significant differences in incidence among different regions (2).

MPP can affect children of all ages, mainly school-aged children and adolescents, and the number of infantile infections has also increased in recent years (3, 4). Most children with MPP have a short course of disease, mild symptoms, no obvious complications and a good prognosis. However, with the significant increase in MP-resistant strains, the proportion of patients who progress to Severe *Mycoplasma pneumoniae* pneumonia (SMPP) due to untimely treatment has risen significantly (5–7).

Children with SMPP have severe clinical symptoms, often presenting with high fever with a long duration of fever, a high proportion of fever, severe cough, listlessness, shortness of breath, dyspnea, etc. These symptoms usually last for 2–3 weeks (7). In addition, SMPP is prone to pulmonary complications such as atelectasis, pleural effusion, and plastic bronchitis. If not intervened in time, it may progress to serious sequelae such as obliterative bronchiolitis, permanent atelectasis, and bronchiectasis. Studies have confirmed that the clinical manifestations of SMPP are not limited to the respiratory system but can involve multiple systems, mainly manifested as gastrointestinal dysfunction, abnormal cardiopulmonary function, hematopoietic system damage, as well as lesions of the skin, mucous membranes and musculoskeletal system, further increasing the complexity of clinical diagnosis and treatment. If diagnosis and treatment are delayed, it will seriously threaten the health of the child and adversely affect their long-term quality of life (8, 9).

Based on this, this study conducted a retrospective analysis of the clinical data of 500 children with MPP who were hospitalized in the pediatric department of Affiliated Hospital of Yan'an University and Yan'an People's Hospital, aiming to provide evidence for the early identification and timely intervention of SMPP, thereby optimizing the treatment plan, improving the prognosis of the children, and enhancing their quality of life. By comparing the above clinical data of the two groups of children, the independent risk factors of SMPP were systematically analyzed, and a visual nomogram prediction model was constructed to provide a basis for early clinical identification and intervention.

## 2 Materials and methods

### 2.1 Research subjects

**Figure 1:**
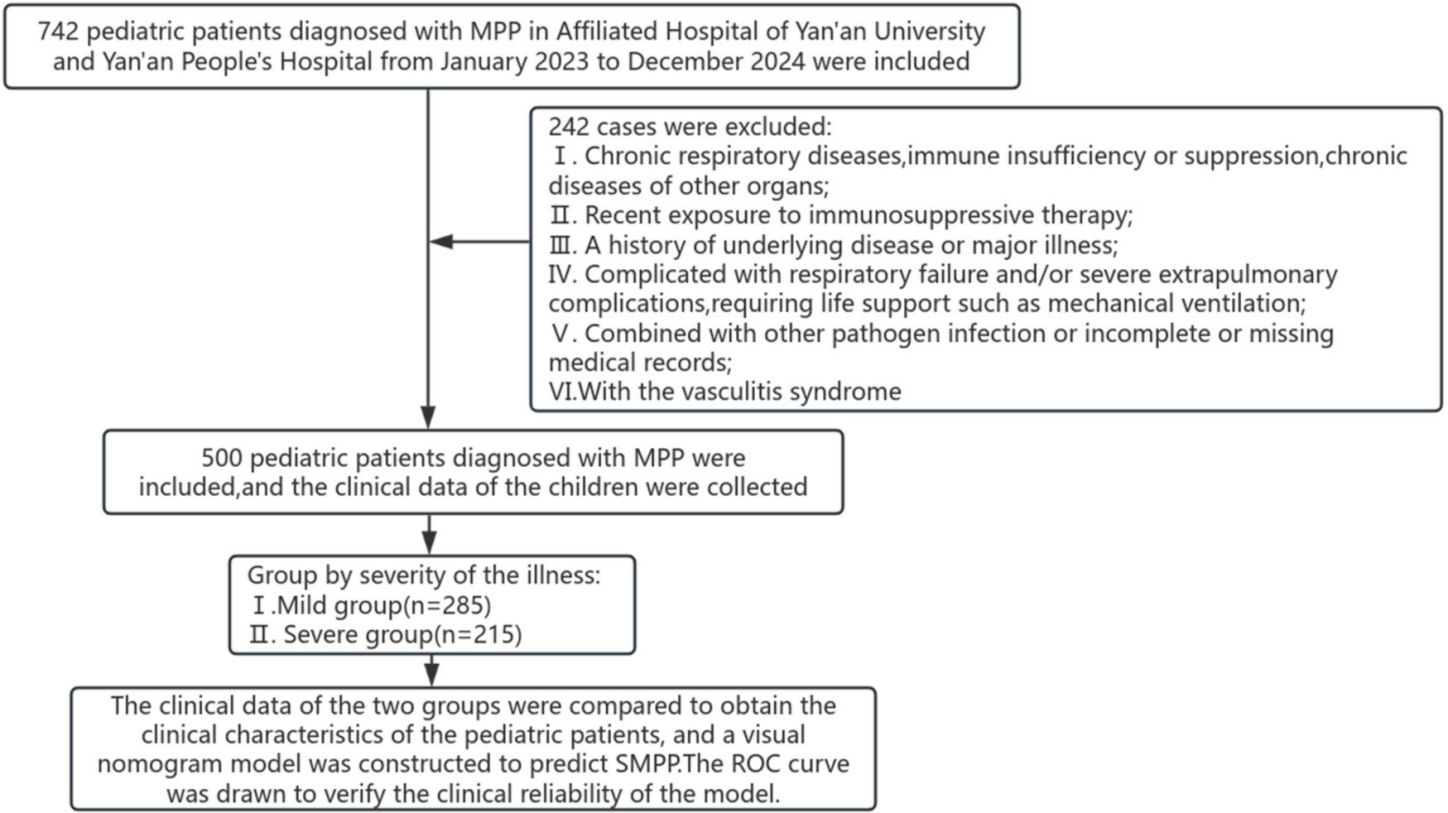
Flow diagram of the study. MPP, *Mycoplasma pneumoniae* pneumonia; SMPP, severe mycoplasma pneumoniae pneumonia.

This retrospective study is based on the clinical data of 500 children with MPP who were hospitalized in pediatrics in Affiliated Hospital of Yan'an University and Yan'an People's Hospital from January 2023 to December 2024. Another 100 children with MPP admitted to the pediatric department of the same hospital from January to March 2025 were selected as an temporal validation cohort (see Figure 1). In this study, children with MPP in the Affiliated Hospital of Yan'an University and Yan'an People's Hospital from January to March 2025 were selected as the time validation cohort. Because the research center is the core institution for the diagnosis and treatment of MPP in pediatrics in Yan'an, no multi-center study has been conducted, so no cross-institution external validation cohort has been set up. Inclusion criteria: I. The diagnosis of MPP was based on the Guidelines for the Diagnosis and Treatment of *Mycoplasma pneumoniae* pneumonia in children (2023 Edition) (8): (i) the presence of respiratory symptoms such as fever and cough, and dry and wet rales could be heard on auscultation of the lungs; (ii) Imaging findings suggest pneumonia changes. Meet the above clinical and imaging manifestations and satisfy one or two of the following conditions: a single serum MP antibody titer ≥1:160, or a fourfold or more increase in double serum MP antibody titers during the course of the disease; MP DNA or RNA test positive. In this study, Mycoplasma pneumoniae was detected using the 13 Respiratory Pathogens Multiplex Detection Kit (Fluorescent PCR-Capillary Electrophoresis) (Health Gene, Cat. No. 1060071). Clinical samples were throat swabs, which were collected and immediately placed in nucleic acid preservation solution for low-temperature transportation to the laboratory. Nucleic acid extraction was performed according to the kit instructions. The purified DNA was used as a template for amplification of the specific target gene of Mycoplasma pneumoniae via multiplex fluorescent PCR-capillary electrophoresis, with RNase P as an internal control to monitor the entire experimental process. After amplification, the PCR products were separated by capillary electrophoresis, and the results were determined based on the specific amplification peaks to achieve qualitative detection of Mycoplasma pneumoniae. II. 2161;. SMPP diagnostic criteria: On the basis of confirmed MPP, according to the diagnostic criteria for severe pneumonia in the diagnosis and treatment of community-acquired pneumonia in children (2019 Edition) (10), a diagnosis can be made if any of the following conditions are present: (I) poor general condition; (ii) Disturbance of consciousness; (iii) Refusal to eat or signs of dehydration; (iv) Significantly increased respiratory rate (≥70 breaths per minute for infants and ≥50 breaths per minute for children over 1 year old); (v) cyanosis; (vi) difficulty breathing (groaning, flapping nostrils, three concave signs); (vii) range of lung infiltration involving multiple lobes or ≥two-thirds of the lungs; (viii) pleural effusion; (ix) have extrapulmonary complications(All the extrapulmonary complications included in this study were mild to moderate, and there were no serious complications requiring life support such as septic shock, cardiogenic shock, acute liver failure, and severe central nervous system involvement (such as encephalitis and convulsion). Such serious cases were excluded according to the exclusion criteria to ensure the consistency of the study subjects); (x) Pulse oxygen saturation ≤0.92. III. Age: ≥1 month, ≤14 years.

Exclusion criteria: (I) those with a history of chronic respiratory diseases, immunodeficiency or suppression of the immune system, or chronic diseases of other organs; (ii) Those who have received immunosuppressant therapy recently; (ii) Those with a history of underlying diseases or who have suffered from major diseases; (iv) Critically ill children (such as those with respiratory failure and/or life-threatening severe extrapulmonary complications requiring mechanical ventilation and other life support); (v)After PCR and Bacterial Blood cell culture, the enrolled children were tested for other respiratory pathogens, including common respiratory viruses (influenza virus, respiratory syncytial virus, adenovirus, etc.), bacteria (Streptococcus pneumoniae, Haemophilus influenzae, etc.), chlamydia, and those who were infected with any of the above pathogens or with incomplete or missing medical records; (vi) those with vasculitis syndrome.

### 2.2 Grouping

They were divided into the mild group (*n* = 285) and the severe group (*n* = 215) based on the severity of the illness; Divided by age into four stages: infancy (1 month −3 years), preschool (3–6 years), school age (6–11 years), and adolescence (≥11 years); By season: Spring (March to May), Summer (June to August), fall (September to November), and winter (December to February).

### 2.3 Clinical data collection

Systematically collect clinical data such as demographic characteristics, symptoms and signs, extrapulmonary comorbidities, laboratory test results, imaging features (All enrolled children underwent chest computed tomography (CT). The clinical indications for chest CT examination in this study were formulated in accordance with the Guidelines for the Diagnosis and Treatment of Mycoplasma pneumoniae pneumonia in children (2023 Edition), including: (1) persistent fever (>3 days) and severe cough unresponsive to conventional anti-infection treatment; (2) abnormal lung auscultation findings (e.g., low breath sounds, fixed rales) with unclear or non-specific chest x-ray results; (3) clinical suspicion of lung consolidation, atelectasis, pleural effusion or other severe pulmonary lesions; (4) need to evaluate the scope and severity of lung lesions for clinical treatment decision-making.), pulmonary function parameters, and bronchoscopic manifestations (Specific indications for bronchoscopy in the mild group included: persistent cough for more than 2 weeks with poor response to conventional treatment, suspected bronchial mucus plug obstruction on chest CT, recurrent wheezing or abnormal small airway function, and unclear etiology of pulmonary infiltration shadows) of the study subjects.

### 2.4 Statistical analysis

Statistical analysis was performed using SPSS 27.0 software. Among the measurement data, those conforming to normal distribution were expressed as mean ± standard deviation (x ± s), and comparisons between groups were conducted using independent sample *t*-tests (for homogeneous variance) or adjusted *t*-tests (for non-homogeneous variance); Non-normally distributed data were expressed as the median (quartile) [M (P_25_, P_75_)] and compared between groups using the Mann–Whitney *U* test. Count data were expressed as frequency (%), and comparisons between groups were conducted using the *χ*^2^ test or Fisher's exact probability method. Multivariate Logistic regression analysis was used to screen for independent risk factors of SMPP, and a visual nomogram prediction model was constructed based on the screening results; The receiver operating characteristic (ROC) curve was plotted to evaluate the predictive power of the model, the calibration curve was used to evaluate the calibration degree of the model, and decision curve analysis (DCA) was used to verify the clinical reliability of the model. A difference was considered statistically significant with a two-sided *P* < 0.05.

## 3 Results

### 3.1 Demographic characteristics

There were 285 cases in the mild group, including 149 males (52.30%) and 136 females (47.70%), with an average age of 6.11 ± 3.17 years. There were 140 school-aged children (49.10%), 119 cases (41.80%) in autumn, and 99 cases (34.70%) in winter. There were 215 severe cases, including 112 boys (52.10%) and 103 girls (47.90%), with an average age of 6.43 ± 3.01 years. Among them, there were 103 school-aged children (47.90%), 72 cases (33.50%) of autumn onset, and 81 cases (37.70%) of winter onset. There were no statistically significant differences in age, gender, school-aged children, or onset season between the two groups (*P* > 0.05) (Table 1).

**Table 1:** Comparison of demographic characteristics between the mild and severe groups of MPP.

Variables	Mild group (*n* = 285)	Severe group (*n* = 215)	*P* value
Age (year)	6.11 ± 3.17	6.43 ± 3.01	0.413
Age group, *n* (%)			0.308
Infancy and early childhood	48 (16.80)	28 (13.10)	
Pre-school age	82 (28.80)	65 (30.20)	
School age	140 (49.10)	103 (47.90)	
Adolescence	15 (5.30)	19 (8.80)	
Gender, *n* (%)			0.967
male	149 (52.30)	112 (52.10)	
female	136 (47.70)	103 (47.90)	
Onset season, *n* (%)			0.278
Spring	22 (7.70)	21 (9.80)	
Summer	45 (15.80)	41 (19.10	
Autumn	119 (41.80)	72 (33.50)	
Winter	99 (34.70)	81 (37.70)	

### 3.2 Clinical manifestations

#### 3.2.1 Fever status

There were 246 cases of fever in the mild group of children, mainly 85 cases (34.50%) with low fever ≤38.0℃ and 133 cases (54.10%) with moderate fever of 38.1℃–39.0℃. The median duration of fever was 5 days. In the severe group, 204 children had fever, mainly 146 (71.60%) with high fever of 39.1℃−42° C, and the median duration of fever was 7 days. There were statistically significant differences in the degree and duration of fever between the two groups (*P* < 0.05) (Table 2).

**Table 2:** Comparison of fever between the mild and severe groups of MPP.

Fever variables	Mild group (*n* = 285)	Severe group (*n* = 215)	*P* value
Degree of fever, *n* (%)			< 0.001
≤38.0 (°C)	85 (34.50)	2 (0.90)	
38.1–39.0 (°C)	133 (54.10)	56 (27.50)	
39. 1–42 (°C)	28 (11.40)	146 (71.60)	
Combined, *n* (%)	246 (86.30)	204 (94.90)	0.006
Duration of fever (day), M(P_25_, P_75_)	5 (3, 6)	7 (5, 9)	<0.001

#### 3.2.2 Respiratory system symptoms and signs

The severe group had a higher incidence of low respiratory sounds, shortness of breath, wheezing, longer duration of cough, and lower blood oxygen saturation at admission, all of which were statistically significant compared with the mild group (*P* < 0.05) (Table 3).

**Table 3:** Comparison of respiratory symptoms and signs between the mild and severe groups of MPP.

Variables	^Mild group (*n*^ ^=^ ^285)^	^Severe group(*n*^ ^=^ ^215)^	*P* value
Cough, *n* (%)	272 (95.30)	200 (93.00)	0.270
Duration of cough (day), M(P25,P75)	12 (10, 13)	15 (14, 18)	<0.001
Panting, *n* (%)	41 (14.40)	68 (31.90)	<0.001
Wheezing, *n* (%)	95 (33.50)	165 (76.80)	<0.001
Rales, *n* (%)	222 (78.10	178 (83.20)	0.157
Low breath, *n* (%)	124 (43.70)	145 (67.40)	<0.001
SPO_2_ (%),M(P_25_, P_75_)	98 (98, 99)	94 (93, 97)	<0.001

#### 3.2.3 Extrapulmonary system complications

There were 66 cases (23.30%) with extrapulmonary complications in the mild group and 109 cases (50.70%) in the severe group, and the difference between the two groups was statistically significant (*P* < 0.05). The incidence of neurological involvement (33 cases, 15.4%) and rash (34 cases, 15.8%) in the severe group was higher than that in the mild group, and the differences were statistically significant (*P* < 0.05) (Table 4).

**Table 4:** Comparison of extrapulmonary system complications between the mild and severe groups of MPP.

Variables	Mild group (*n* = 285)	Severe group (*n* = 215)	*P* value
Extrapulmonary complications, *n* (%)	66 (23.30)	109 (50.70)	<0.001
≥2 systems, *n* (%)	40 (14.00)	80 (37.20)	<0.001
Circulatory system, *n* (%)	22 (7.90)	75 (34.70)	<0.001
Myocardial damage	5 (1.90)	24 (11.20)	<0.001
Arrhythmology	17 (6.00)	51 (23.70)	<0.001
Digestive system, *n* (%)	40 (14.00)	87 (40.70)	<0.001
Nausea and vomiting	24 (8.40)	44 (20.40)	<0.001
Abdominal pain and diarrhea	12 (4.20)	22 (10.20)	0.009
Liver function impairment	4 (1.40)	21 (9.80)	<0.001
Nervous system, *n* (%)	9 (3.30)	33 (15.40)	<0.001
Headache	4 (1.40)	24 (11.60)	<0.001
Blood system, *n* (%)	12 (4.20)	25 (11.60)	0.003
Anemia	4 (1.40)	10 (4.60)	0.083
Elevated platelets	8 (2.80)	15 (7.00)	0.035
Urinary system, *n* (%)	2 (0.90)	5 (2.50)	0.352
Electrolyte imbalance, *n* (%)	5 (1.90)	12 (5.60)	0.059
Rash, *n* (%)	14 (5.10)	34 (15.80)	<0.001

All extrapulmonary complications in this study were mild to moderate, without severe complications requiring life support (e.g., septic shock, cardiogenic shock, acute liver failure, severe central nervous system involvement such as encephalitis and convulsion). Severe extrapulmonary complications were excluded according to the study exclusion criteria.

### 3.3 Laboratory tests

The White blood cells(WBC), Neutrophils, Neutrophil-to-Lymphocyte Ratio(NLR), Platelets, Platelet-to-Lymphocyte Ratio(PLR),Mean Platelet Volume(MPV), C-reactive Protein(CRP), Serum Amyloid A(SAA), Procalcitonin (PCT) and Erythrocyte Sedimentation Rate(ESR) in the severe group were significantly increased, and there were statistically significant differences compared with the mild group (*P* < 0.05). While lymphocyte count and hemoglobin were decreased in the severe group, there were statistically significant differences compared with the mild group (*P* < 0.05). There was no difference in Platelet Distribution Width(PDW) between the two groups (*P* > 0.05);The levels of Lactate Dehydrogenase(LDH), Alanine Aminotransferase(ALT), Aspartate Aminotransferase(AST), Creatinine(Cr), Creatine Kinase(CK), Creatine Kinase-MB Isoenzyme (CK-MB),Mycoplasma Pneumoniae Antibody Titer(MP antibody titers), D-dimer, Prothrombin Time(PT) and Fibrinogen(FFIB) in the severe group were significantly higher than those in the mild group, and the albumin level was lower than that in the mild group, and the differences were statistically significant (*P* < 0.05) (Table 5).

**Table 5:** Comparison of laboratory test indicators between the mild and severe groups of MPP.

Variables	Mild group (*n* = 285)	Severe group (*n* = 215)	*P* value
White blood cell count, ×10^9^/L	7.62 (6.45, 8.87)	8.14 (6.06, 10.50)	0.034
Neutrophils count, ×10^9^/L	3.79 (2.48, 5.18)	4.99 (3.27, 7.60)	<0.001
Lymphocytes count, ×10^9^/L	2.55 (1.95, 3.79)	2.23 (1.57, 2.84)	<0.001
NLR	1.63 (0.68, 2.47)	2.36 (1.52, 3.27)	<0.001
Hemoglobin, g/L	126 (118, 132)	115 (107, 129)	<0.001
Platelets count, ×10⁹/L	324 (252, 378)	379 (260, 487)	<0.001
MPV, fL	9.3 (8.9, 10.0)	9.6 (9.1, 10.2)	<0.004
PDW, fL	10.9 (9.4, 11.6)	10.7 (9.2, 12.3)	0.885
PLR	120.35 (96.37, 164.94)	161.74 (122.43, 222.37)	<0.001
CRP, mg/L	10 (10, 13.74)	19 (10, 47.75)	<0.001
SAA, mg/L	34.5 (25.10, 37.50)	47 (35.60, 103.22)	<0.001
ESR, mm/h	35 (25, 35)	65 (45, 77)	<0.001
PCT, μg/L	0.1 (0.05, 0.25)	1.15 (0.25, 2.05)	<0.001
ALB, g/L	42.50 (40.30, 46.60)	39.10 (38.10, 40.15)	<0.001
LDH, U/L	291 (262, 342)	374 (302, 457)	<0.001
ALT, U/L	23 (19, 31)	44 (23, 53)	<0.001
AST, U/L	31 (25, 36)	43 (26, 49)	0.001
Cr, μmol/L	31 (24, 38.9)	34 (29.75, 41.6)	<0.001
BUN, μmol/L	3.8 (3.0, 4.4)	3.5 (2.6, 4.8)	0.376
CK, U/L	78 (54, 125)	94 (58, 155)	0.007
CK-MB, ng/mL	3.3 (2.3, 4.2)	3.6 (2.7, 4.7)	<0.001
D-dimer, mg/L	0.65 (0.56, 0.79)	0.89 (0.64, 1.02)	<0.001
PT, s	11.60 (11.10, 11.90)	12.50 (12.10, 13.20)	<0.001
FIB, g/L	3.87 (3.12, 4.11)	4.57 (4.08, 5.21)	<0.001
APTT, s	27.50 (25.70, 28.90)	27.80 (26.10, 29.20)	<0.068
MP antibody titer	320 (160, 320)	640 (320, 640)	<0.001

NLR, neutrophil-to-lymphocyte ratio; MPV, mean platelet volume; PDW, platelet distribution width; PLR, platelet-to-lymphocyte ratio; CRP, C-reactive protein; SAA, serum amyloid A; ESR, erythrocyte sedimentation rate; PCT, procalcitonin; ALB, albumin; LDH, lactate dehydrogenase; ALT, alanine aminotransferase; AST, aspartate aminotransferase; Cr, creatinine; BUN, urea nitrogen; CK, creatine kinase; CK-MB, creatine kinase-MB isoenzyme; PT, prothrombin time; FIB, fibrinogen; APTT, activated partial thromboplastin time; MP antibody titer, *Mycoplasma Pneumoniae* antibody titer. *p* values were calculated using the Mann–Whitney *U* test for continuous variables and chi-square test for categorical variables. Variables with *p* < 0.05 were considered statistically significant.

### 3.4 Imaging examination results

In the mild case group, lesions in the lower lobe of the left lung (59 cases, 20.70%) and patchy/ground-glass shadows (255 cases, 89.80%) were predominant; Severe cases were dominated by right lower lobe lesions (56 cases, 26.00%), large patchy shadows (56 cases, 19.60%), and patchy shadows (67 cases, 31.10%), with higher incidences of lung consolidation (114 cases, 52.80%), atelectasis (43 cases, 20.00%), and pleural effusion (22 cases, 10.20%). There were statistically significant differences between the two groups in terms of lesion location, lesion range, and the incidence of lung consolidation, atelectasis, and pleural effusion (*P* < 0.05) (Table 6).

**Table 6:** Comparison of imaging manifestations between the mild and severe groups of MPP.

Variables	Mild group (*n* = 285)	Severe group (*n* = 215)	*P* value
Lesion site, *n* (%)			0.013
Upper lobes of both lungs	46 (16.20)	16 (7.40)	
Lower lobes of both lungs	46 (16.20)	46 (21.40)	
Upper lobe of the right lung	37 (13.00)	30 (14.00)	
Middle lobe of the right lung	10 (3.30)	6 (2.80)	
Lower lobe of the right lung	49 (17.20)	56 (26.00)	
Upper lobe of the left lung	38 (13.30)	22 (10.20)	
Lower lobe of the left lung	59 (20.70)	39 (18.20)	
Lesion range, *n* (%)			<0.001
Large patches	6 (1.80)	56 (19.60)	
Sheet-like	24 (8.40)	67 (31.10)	
Patchy/frosted glass shadows	255 (89.40)	92 (42.80)	
Lung consolidation, *n* (%)	28 (9.80)	114 (52.80)	<0.001
Atelectasis, *n* (%)	3 (1.05)	43 (20.00)	<0.001
Pleural effusion, *n* (%)	3 (1.05)	22 (10.20)	<0.001
Thickened pleura, *n* (%)	1 (0.35)	6 (2.80)	0.118
Enlarged hilar lymph nodes, *n* (%)	2 (0.70)	8 (3.70)	<0.09

All children enrolled in this study underwent chest computed tomography (CT) examination, and chest x-ray was used as a supplementary imaging method. All radiological findings in this table were based on chest CT results, which could accurately identify subtle lung lesions such as lung consolidation, mild atelectasis and small amount of pleural effusion.

### 3.5 General pulmonary function and bronchoscopic performance results

Pulmonary function tests were completed in 261 children over 6 years old. Indicators such as Forced vital capacity (FVC), Forced expiratory volume in one second/forced vital capacity (FEV1/FVC), Peak expiratory flow (PEF), Maximum mid-expiratory flow rate (MMEF), Flow rate at 25% forced vital capacity (FEF25), Flow rate at 50% forced vital capacity (FEF50), Flow rate at 75% forced vital capacity (FEF75) in the severe group were significantly lower than those in the mild group (*P* < 0.05) (Table 7).

**Table 7:** Comparison of general pulmonary function between the mild and severe groups of MPP.

Variables	Mild group (*n* = 116)	Severe group (*n* = 145)	*P* value
FEV1/FVC (%)	94.40 (93.50, 96.40)	89.70 (86.50, 93.30)	<0.001
FVC	96.50 (83.80, 100.90)	77.40 (66.80, 93.70)	<0.002
PEF	82.90 (77.30, 91.90)	80.40 (69.80, 87.40)	<0.003
FEF25	76.30 (66.90, 94.60)	68.80 (57.60, 90.30)	<0.002
FEF50	73.90 (67.80, 82.50)	63.40 (47.20, 80.70)	<0.001
FEF75	63.20 (52.00, 72.50)	46.70 (40.70, 61.00)	<0.001
MMEF	73.70 (65.80, 85.90)	62.30 (35.90, 60.20)	<0.001

Non-normal distribution data were expressed as median (quartile), M (P_25_, P_75_).

Among 322 children who underwent bronchoscopy, mucosal congestion and edema were the most common manifestations (102 cases, 94.40%); The incidence of mucus thrombus, shaped sputum thrombus, inflammatory stenosis and mucosal erosion in the severe group was significantly higher than that in the mild group (*P* < 0.05), while there was no statistically significant difference in mucosal nodular protrusion and mucosal congestion and edema between the two groups (*P* > 0.05) (Table 8).

**Table 8:** Comparison of bronchoscopic manifestations between the mild and severe groups of MPP.

Variables	Mild group (*n* = 109)	Severe group (*n* = 213)	*P* value
Mucus plug	15 (14.40)	107 (50.70)	<0.001
Shaping sputum suppositories	1 (0.90)	23 (10.50)	<0.001
Inflammatory stenosis of the mucosa	6 (5.10)	83 (39.30)	<0.001
Mucosal erosion	3 (2.70)	64 (30.20)	<0.001
Mucosal nodular protrusions	33 (29.80)	83 (39.30)	0.145
Mucosal congestion and edema	102 (94.40)	207 (97.50)	0.07

Bronchoscopy for children with mild MPP was a clinically indicated selective examination rather than a routine test. The implementation was based on specific clinical or radiological evidence, and all procedures were performed in accordance with the Guideline of Pediatric Flexible Bronchoscopy in China (2018 Version) with no severe complications occurred.

### 3.6 Analysis of risk factors for SMPP and construction of predictive models

#### 3.6.1 Binary logistic regression analysis of SMPP risk factors

Binary Logistic regression analysis was used in the study, with disease severity (mild = 0, severe = 1) as the dependent variable, and indicators with significant differences between groups in the univariate analysis were included as independent variables. Assign values to categorical variables (fever: none = 0, with = 1; wheezing, shortness of breath, low breathing sound: none = 0, with = 1; extrapulmonary complications, lung consolidation, atoptasis, pleural effusion, hilar lymph node enlargement: none = 0, with = 1; lesion range: patchy/ground-glass-like = 0, patchy = 1, large patchy = 2; Mucus plugs, shaped sputum plugs, inflammatory stenosis of the mucosa, mucosal erosion: none = 0, with = 1) were included in the binary Logistic regression equation. The results showed: ESR[OR = 1.101(95%CI: 1.033, 1.173)], LDH[OR = 1.009(95%CI: 1.001, 1.016)], lung consolidation [OR = 4.932(95%CI: [1.234, 19.566] and D-dimer [OR = 1.009(95%CI: 1.213, 6.208)] were risk factors for SMPP (*P* < 0.05), and albumin [OR = 0.784(95%CI: 0.624, 0.985)] was a protective factor for SMPP (*P* < 0.05) (Table 9).

**Table 9:** Binary logistic regression analysis of SMPP risk factors.

Variables	*β*	Standard error	OR	95%CI	*P* value
ESR, mm/h	0.096	0.032	1.101	[1.033, 1.173]	0.003
ALB, g/L	−0.243	0.116	0.784	[0.624, 0.985]	0.036
LDH, U/L	0.009	0.004	1.009	[1.001, 1.016]	0.027
Lung consolidation	1.596	0.703	4.932	[1.243, 19.566]	0.023
D-dimer, mg/L	1.009	0.417	2.744	[1.213, 6.208]	0.015

OR, odds ratio; CI, confidence interval; variables with *p* < 0.05 were considered statistically significant.

#### 3.6.2 ROC curve analysis

**Figure 2:**
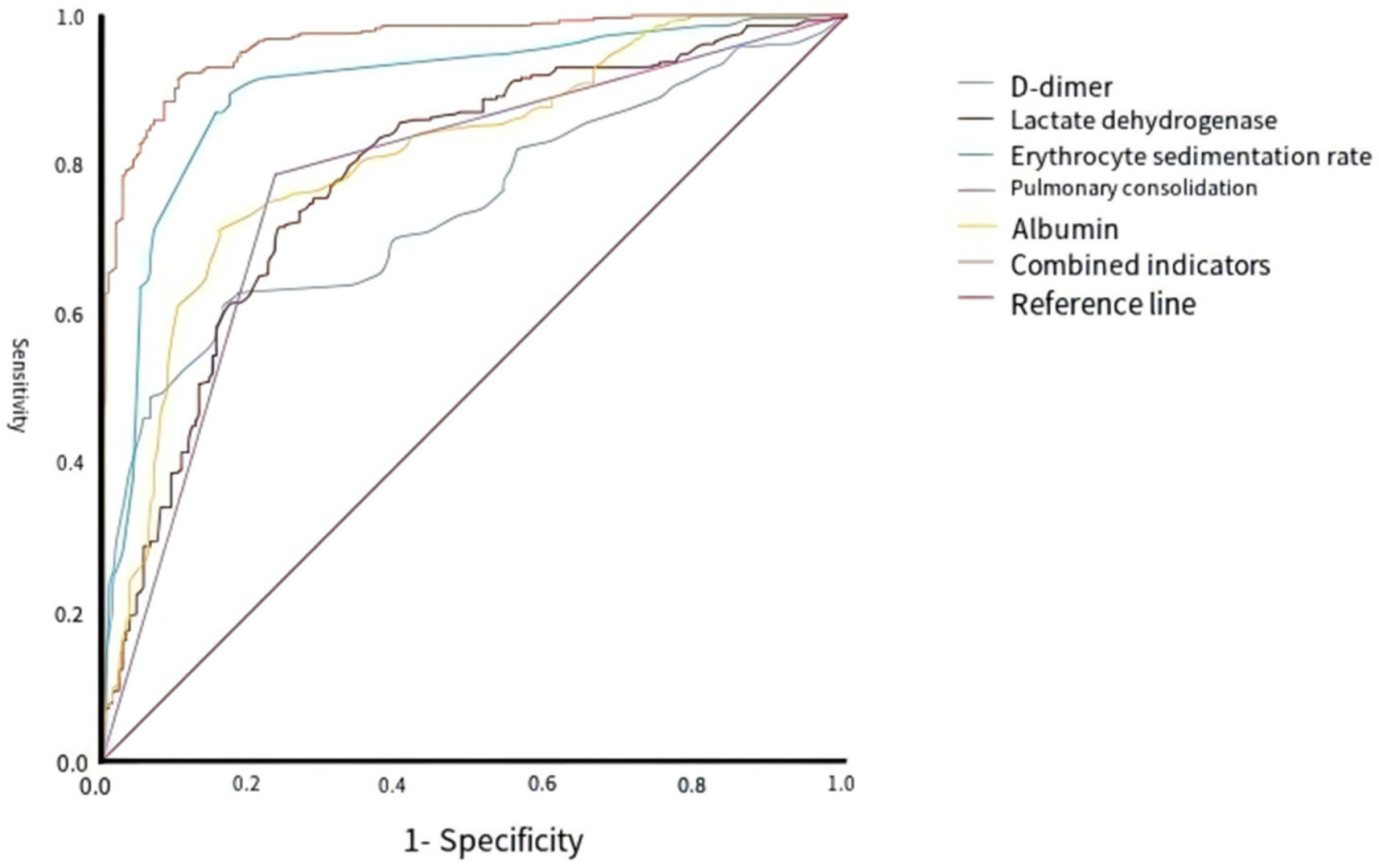
ROC curve analysis of each risk factor and combined indicators.

Receiver operating characteristic curve analysis was performed on ESR, ALB, LDH, lung consolidation and D-dimer after controlling for confounding factors. The results showed that the diagnostic efficacy ranking of each indicator (indicated by AUC) was ESR (0.86) >ALB (0.82) >LDH (0.78) >lung consolidation (0.75) >D-dimer (0.72). The cut-off values were ESR 40.5 mm/h, ALB 39 g/L, LDH 345 IU/L, and D-dimer 0.91 mg/L, respectively. (Lung consolidation is a categorical variable, none = 0, yes = 1, no specific cutoff value is set) (Table 10; Figure 2).

**Table 10:** ROC curve analysis of each risk factor for SMPP.

Variables	Cut-off value	AUC	Standard error	Youden Index	Sensitivity	Specificity
ESR	40.5	0.902	0.022	0.723	0.895	0.828
Albumin	39	0.811	0.020	0.616	0.774	0.842
LDH	345	0.787	0.021	0.545	0.782	0.763
Lung consolidation	–	0.777	0.022	0.553	0.786	0.767
D-dimer	0.91	0.742	0.015	0.727	0.792	0.935

The cut-off value of each detection index was determined based on the value corresponding to the maximum value of the Youden index (sensitivity + specificity −1).

#### 3.6.3 Construction and validation of the nomogram model

**Figure 3:**
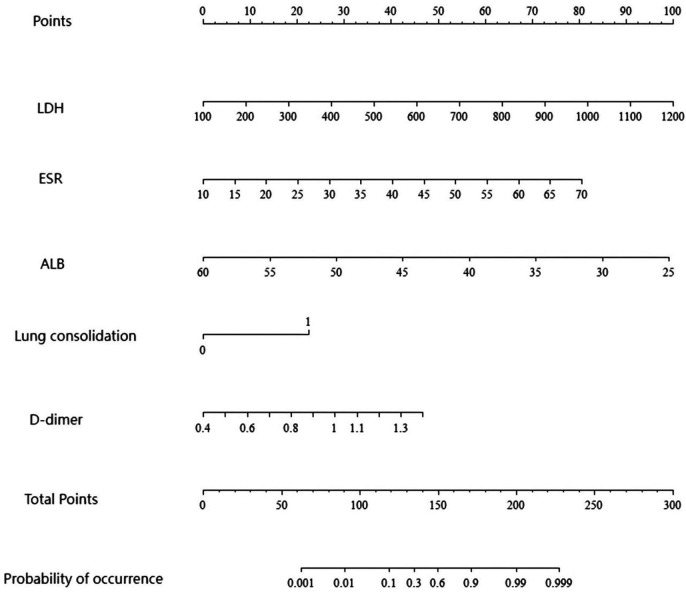
Nomogram prediction model of SMPP.

Incorporating risk factors into the model yields the SMPP nomogram prediction model (Figure 3), LDH ≈ 22.5 points, ESR ≈ 40.5 points, albumin ≈ 57.5 points, D-dimer ≈ 23.5 points, lung consolidation ≈ 22 points, total score ≈ 22.5 + 40.5+57.5 + 23.5 + 22 = 166 points, P (SMPP) ≈ 80%–90%. The model is validated both internally and temporally. For internal validation, the data included in the model is self-weighted by Bootstrap sampling and substituted into the model for validation; Temporal validation was conducted by selecting 100 inpatients with MPP in our hospital.

**Figure 4:**
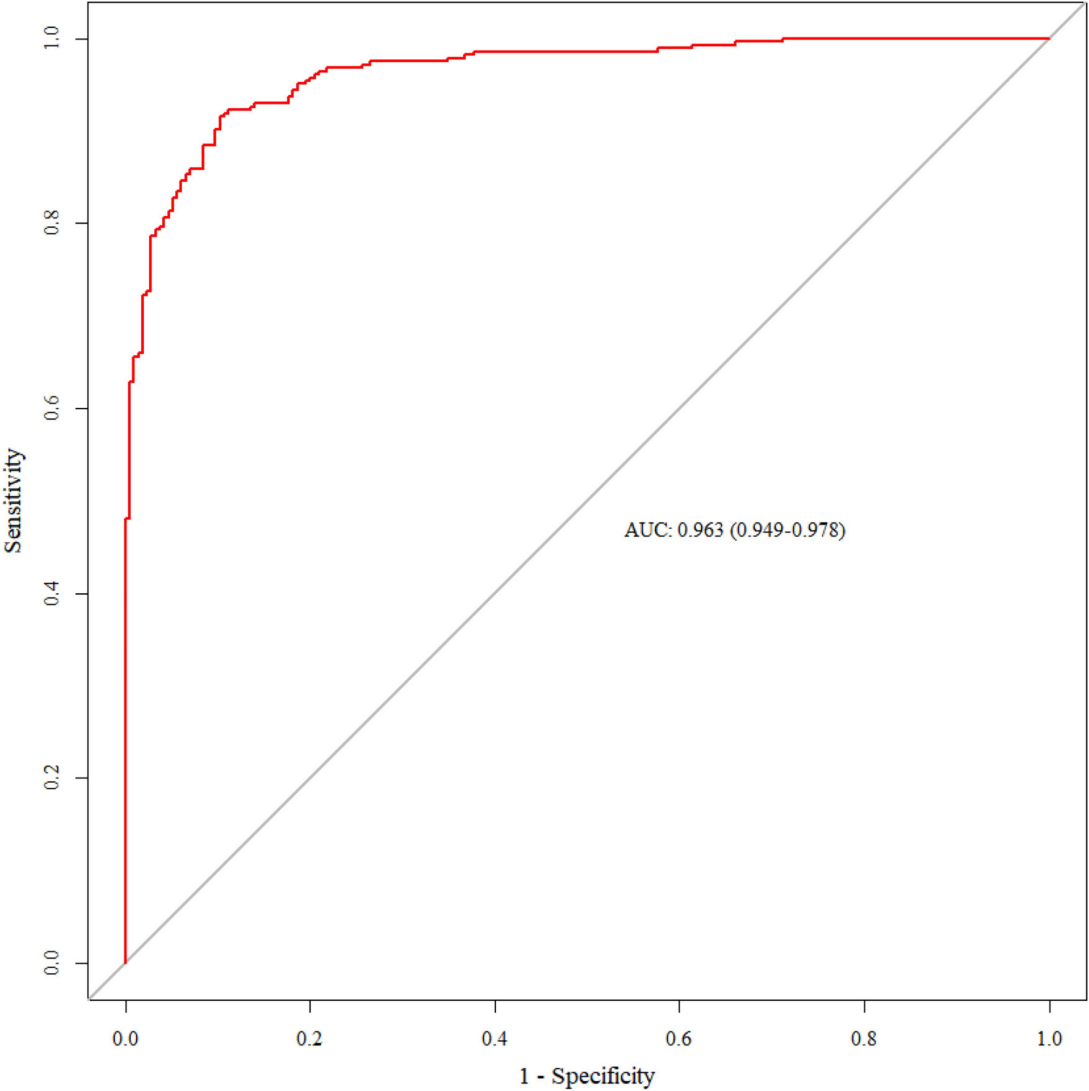
ROC curve for internal validation of the SMPP nomogram model.

**Figure 5:**
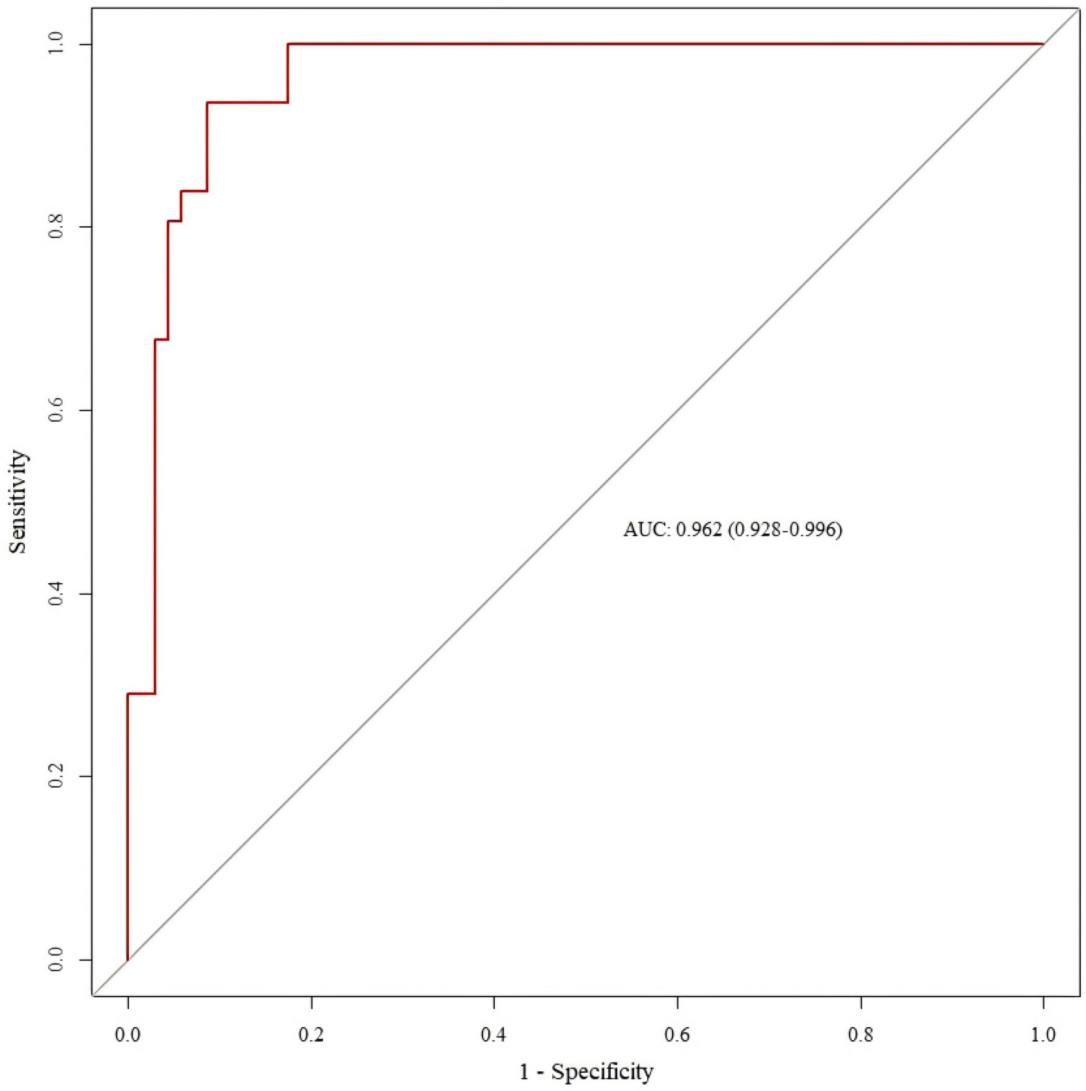
ROC curve for external validation of the SMPP nomogram model.

The results showed that the internal validation AUC was 0.963 (95%CI: 0.949, 0.978) (Figure 4), and the temporal validation AUC was 0.962 (95%CI: 0.928, 0.996) (Figure 5).

**Figure 6:**
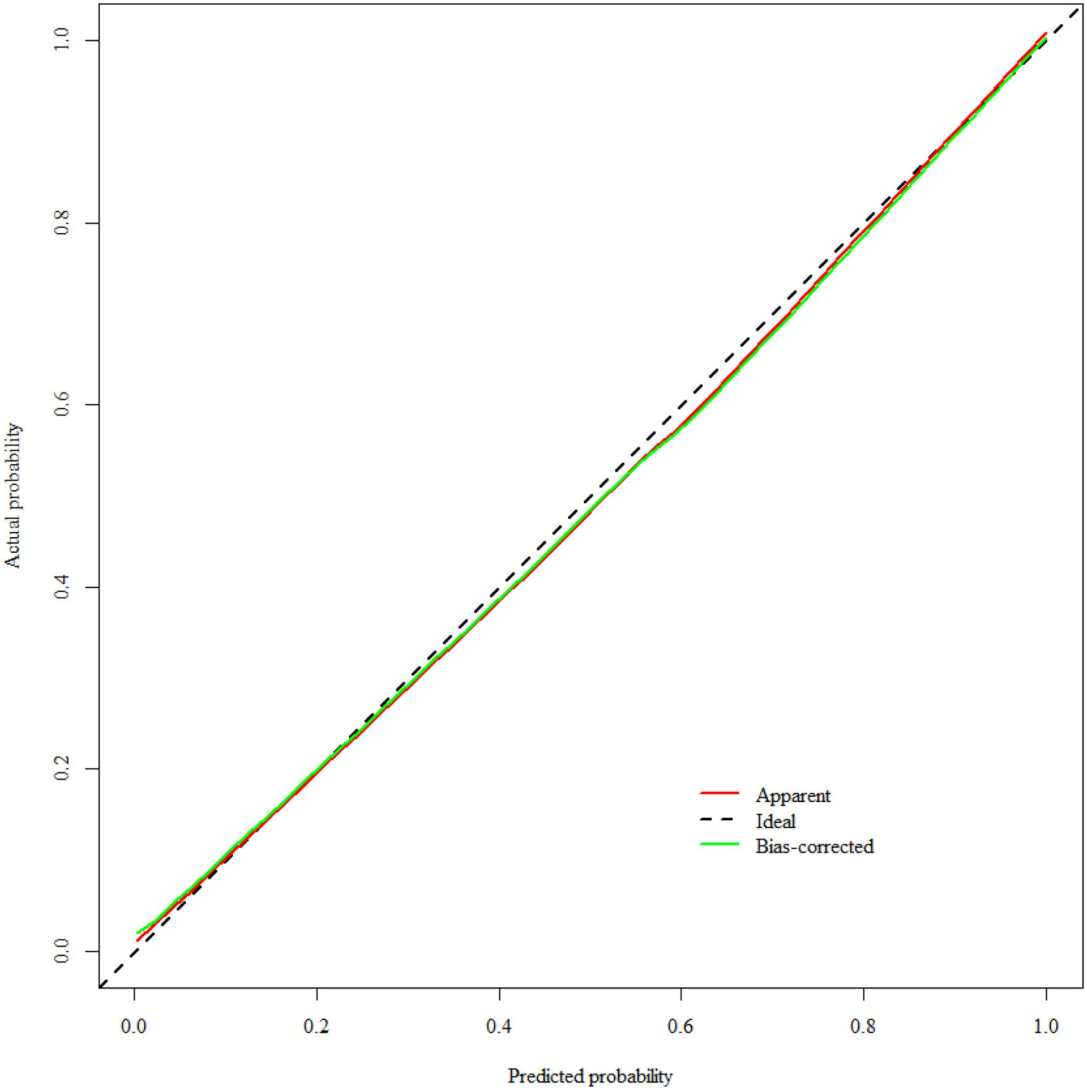
Calibration curve of the SMPP nomogram prediction model.

**Figure 7:**
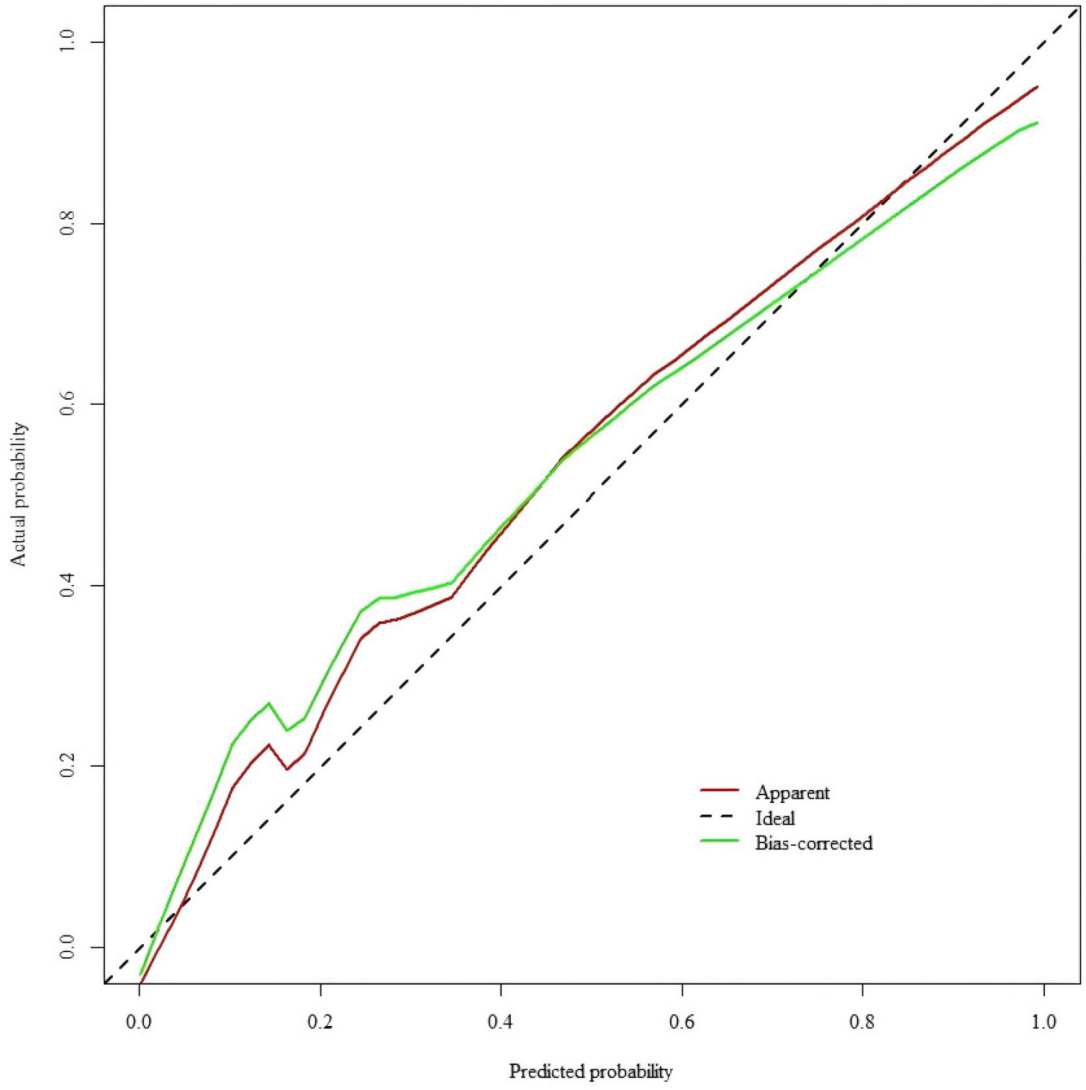
Calibration curve for temporal validation of the prediction model.

The Hosmer-Lemeshow test results showed that *X^2^* = 11.458, *df* = 8, *p* = 0.1771(>0.05). After resampling and adjusting the data using the Bootstrap method, the calibration curve of the model was plotted with a high degree of conformity to the ideal curve (Figure 6), indicating good calibration of the model. The temporal validated calibration curve, as shown in Figure 7.

**Figure 8:**
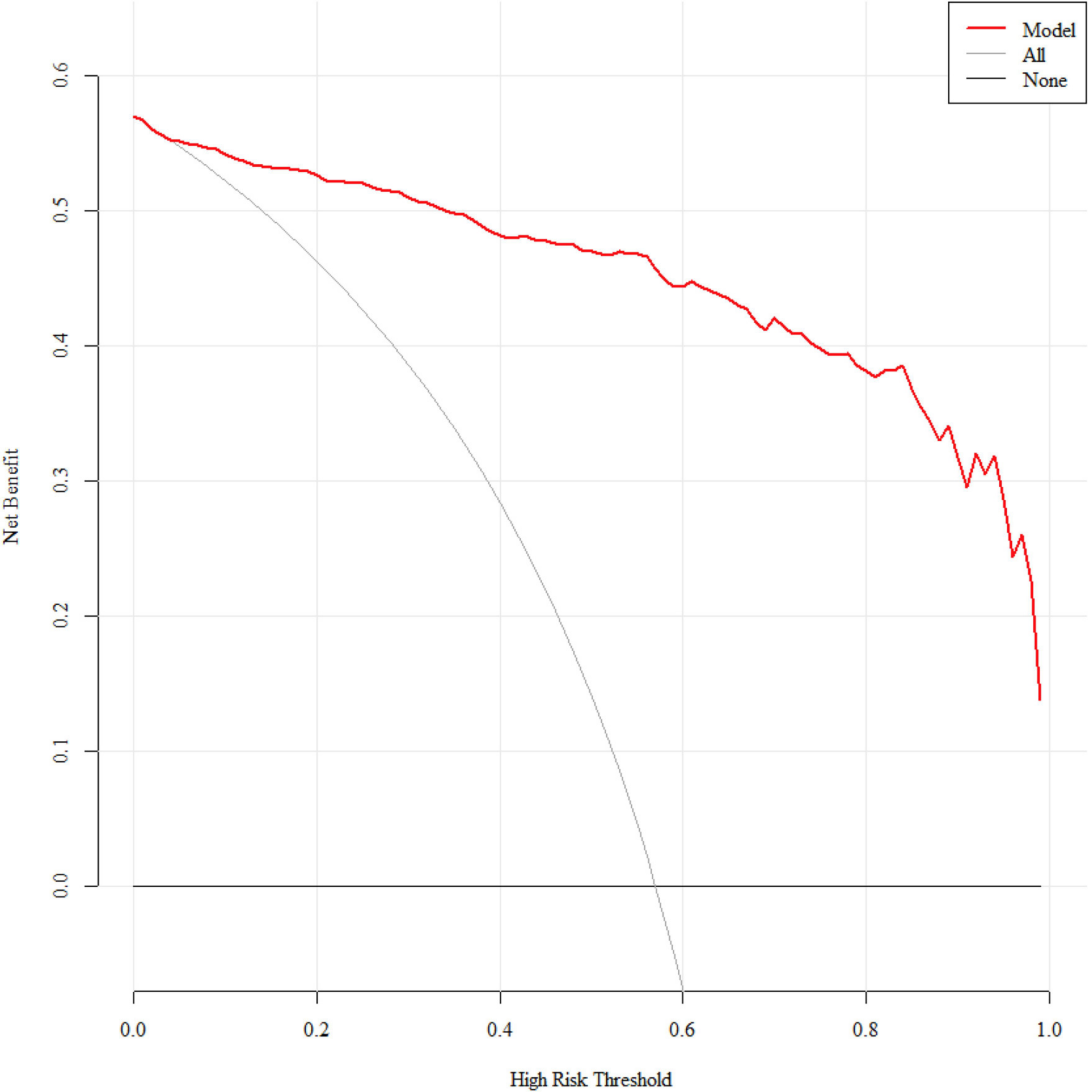
Decision curve of the SMPP nomogram prediction model.

Internal validation decision curve analysis showed that the net benefit rate for predicting SMPP was greater than 0 and clinically significant when the threshold probability was 4%–99% (Figure 8).

**Figure 9:**
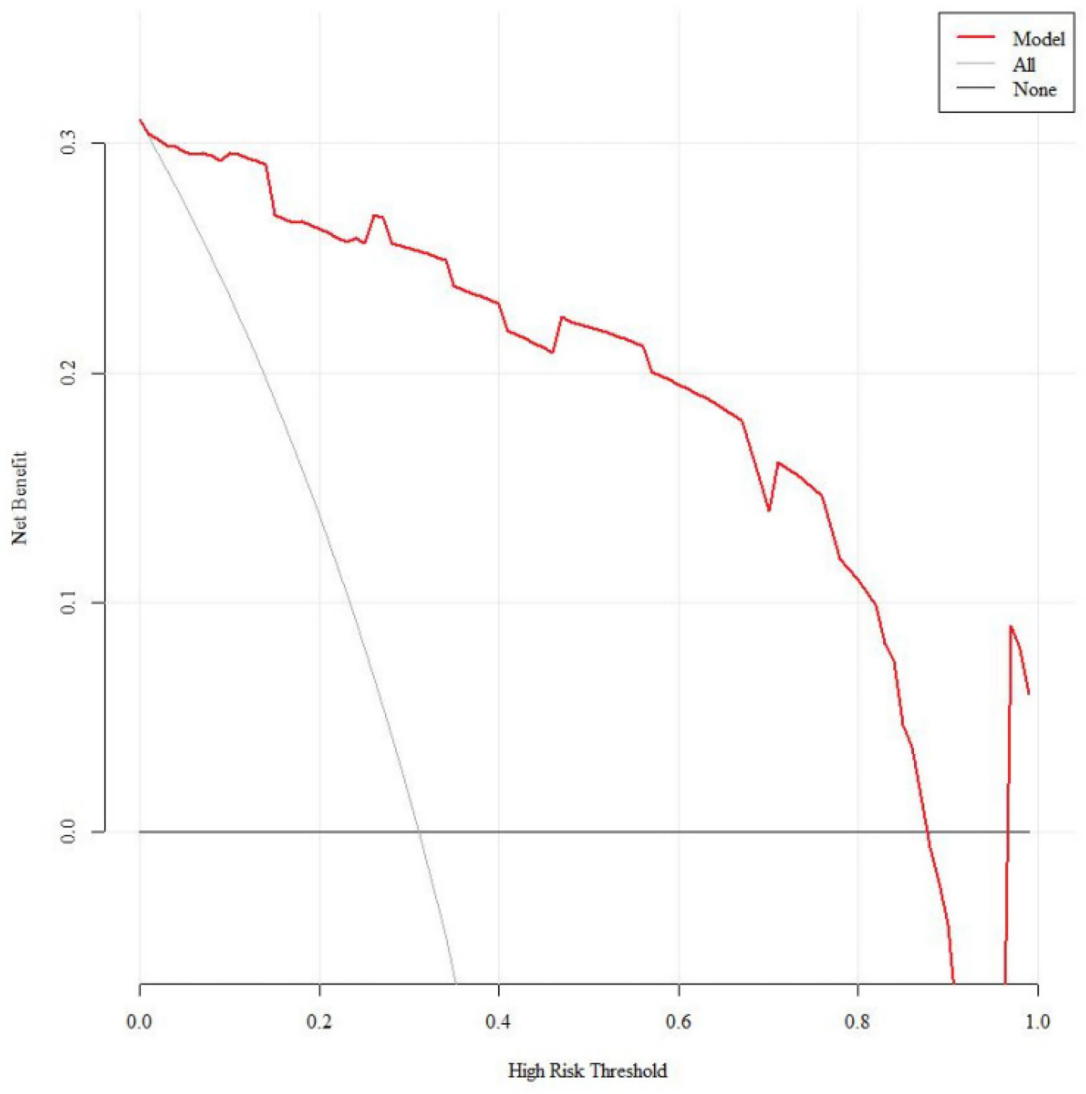
Decision curves for temporal validation of the SMPP nomogram prediction model.

Temporal validation decision curve analysis showed that the net benefit rate for predicting SMPP was greater than 0 and clinically significant when the threshold probabilities were 1%–87% and 97%–99% (Figure 9).

## 4 Discussion

### 4.1 General situation analysis

MP, as an important pathogenic microorganism of respiratory tract infections in children, is characterized by a periodic outbreak trend, with an epidemic interval of approximately 3–7 years, and the high-incidence population is concentrated in school-age children and adolescents (aged ≥6 years) (11). The study shows that the highest prevalence of MP infection was among school-aged children, followed by preschool and early childhood, which is in line with the characteristic that MP infection occurs in all age groups in China, with school-aged children being the majority (11, 12). Previous studies have shown that MP infections have not only seasonal differences but also significant regional variations (13). The two groups of children with MPP in this study mostly developed the disease in autumn and winter, which is the same as previous research conclusions (14). Contrary to the research report of Liu et al. (10), the onset season of children with MPP was more common in summer, mainly considering that different regions, different climatic temperatures and humidity can affect the transmission of the MP pathogen, resulting in different research results. However, in terms of gender, the ratio of male to female in both groups was comparable, indicating that there was no significant association between the severity of the disease and gender, which is consistent with previous studies (5).

### 4.2 Analysis of clinical manifestations

In this study, 90% of the children with MPP had fever, with high fever being predominant in the severe group. The degree and duration of fever were higher than those in the mild group, consistent with the results of previous studies on high fever and long duration of fever in children with severe MPP (15). The duration of cough in the severe group was longer than that in the mild group, and the incidence of wheezing, shortness of breath, and low auscultation of breath sounds was higher than that in the mild group, as confirmed by Wang et al. (16). The study found that although children in the severe group did not reach the diagnostic threshold for severe pneumonia in early SpO_2_ monitoring, their blood oxygen saturation decreased compared with children in the mild group, and the difference was significant (*P* < 0.05). SpO_2_ can indirectly reflect the degree of lung tissue damage, providing an important reference basis for clinical judgment of the severity of the disease and treatment effect (8).

Through comparative analysis between groups, it was found that the incidence of extrapulmonary complications in children with severe MPP was significantly increased, which is consistent with the conclusion of Gong et al. (15) that extrapulmonary complications are risk factors for SMPP. The incidence of extrapulmonary system damage in the severe group showed obvious organ-specific distribution characteristics, with digestive system involvement being the most common, followed by circulatory system damage. The conclusion of Liu et al. (17) also indicated that in Chinese SMPP children, the most obvious extrapulmonary manifestation was digestive system organ involvement, accounting for about one-third, followed by cardiovascular system, accounting for about one-fourth. The results of this study indicated that nausea and vomiting were most common in the digestive system of the severe group, while in the research results of Yao et al. (18) in China, liver function impairment was the most common digestive system impairment in SMPP. Currently, there are inconsistent reports on the main manifestations of the digestive system, which may be related to the age of the research subjects. The younger the age, The less developed digestive and immune systems, the direct action of pathogens, systemic inflammatory responses, and side effects of drugs can all lead to gastrointestinal symptoms in children.

### 4.3 Laboratory-related inspection and analysis

In this study, the White blood cell count, Neutrophil count, NLR, Platelet count, PLR, and CRP in the mild group were all lower than those in the severe group (*P* < 0.05), which is consistent with previous research results (19–21). The hemoglobin levels in the mild group were higher than those in the severe group (*P* < 0.05), which might be related to the elevated levels of inflammatory factors in the severe group, which inhibited the production of Erythropoietin (EPO) and the response of bone marrow to EPO, affecting the production of hemoglobin. Recent findings by Xu et al. (22) also suggest that in children with more severe MPP and poorer prognosis, the decrease in hemoglobin is more pronounced. The PCT values in the mild group were lower than those in the severe group (*P* < 0.05). Pan et al. (23) indicated that PCT expression was highly expressed in the serum of children with severe MPP, mainly due to severe infection causing excessive pro-inflammatory factors in the body and disrupting the immune system. Excessive immune responses in children with severe MPP can cause an increase in PCT expression. Meanwhile, in a Mate (24) report on RMPP, PCT was identified as one of the most reliable predictors of RMPP.

CRP, SAA, PCT, ESR and other inflammatory markers were included in the univariate analysis, which showed a significant correlation with SMPP (*P* < 0.05). After adjusting for confounding factors by multivariate binary Logistic regression analysis, ESR was the only independent risk factor for SMPP (OR = 1.101, 95%CI: 1.033–1.173, *P* = 0.003), and the other inflammatory indicators were gradually removed from the model due to the collinearity with other variables (for example, the collinearity coefficient between CRP and SAA >0.7). In addition, ROC curve analysis showed that the AUC of ESR for SMPP diagnosis was 0.902, which was higher than CRP (AUC = 0.725), PCT (AUC = 0.768) and other indicators. ESR was a clinical routine and low-cost detection index, which was suitable for primary medical institutions, so it was finally included in the prediction model. This is similar to the conclusion of Gordon O et al. (25), while the critical value of ESR as an independent risk factor for SMPP in the study by Liu et al. (10), was 36.5 mm/h. Although there were some differences in the specific cut-off values, all studies confirmed that ESR could serve as an important reference indicator for assessing the severity of *mycoplasma pneumoniae* pneumonia.

The results of this study indicated that the serum LDH levels of children with SMPP were significantly elevated and were significant in the multivariate analysis. When the cut-off value of LDH was set at 345 U/L, the diagnostic specificity for SMPP was 76.30% and the sensitivity was 71.60%. This is similar to the conclusion of Liu et al. (26), but in other (27) study results, LDH ≥ 337.38 U/L often indicates that the disease may develop towards SMPP. Therefore, more studies are needed to confirm the optimal cut-off value of LDH for predicting the occurrence of SMPP.

The study found that serum ALB levels were lower in the severe group compared with children with mild symptoms, and multivariate regression analysis showed that albumin levels were an independent protective factor for SMPP in children. In a retrospective study by Cheng et al. (28), LDH, ALB, Neutrophil ratio, and high fever were identified as predictive values for RMPP, and their clinical value was verified through decision curve analysis and clinical impact curves. At present, there are few studies on the relationship between SMPP and albumin, and a large number of studies are needed to further confirm that albumin is an independent protective factor for SMPP.

Multivariate analysis of this study suggests that D-dimer is an independent risk factor for predicting SMPP. ROC curve analysis showed that when the cut-off value of D-dimer was 0.91 mg/L, the predictive sensitivity for SMPP was 48.80% and the specificity was 93.50%, which was consistent with previous research conclusions. Furthermore, Wen J et al. (29) found that D-dimer was an independent risk factor for RMPP, and the ROC curve showed a cut-off value of 2.1 mg/L. Due to the limited number of studies, a large number of clinical studies are still needed in the future to evaluate the application value of D-dimer in predicting SMMP and the optimal cut-off value.

### 4.4 Analysis of imaging manifestations

The imaging manifestations of MPP are heterogeneous. Common signs mainly include large patchy or focal shadows in the lungs, increased density in the hilar region, patchy or cloud-like infiltration shadows, etc. Some cases may also present special imaging features (30). In this study, there were 142 cases (28.40%) of MPP children with pulmonary consolidation, 46 cases (9.20%) of atelectasis, 25 cases (5.00%) of pleural effusion, 7 cases (1.40%) of pleural thickening, and 10 cases (2.00%) of hilar lymph node enlargement. The proportion of children with pulmonary consolidation, atoptasis, pleural effusion and hilar lymph node enlargement in the severe group was significantly higher than that in the mild group, and there was a significant difference between the two groups (*P* < 0.05), mainly related to the strong immune response and inflammatory response in children with SMPP, consistent with the conclusion of previous studies (28). In this study, the lung lesions in the severe group were mainly large plate-like/patchy, while those in the mild group were mainly patchy. There was a significant difference in the lesion range between the two groups (*P* < 0.05), and the study conclusion was consistent with that of Gao et al. (31), indicating that when the lung lesions in children with MPP are extensive and the degree of injury is severe, Clinicians should be highly vigilant about the possibility of progression to SMPP.

### 4.5 Pulmonary function manifestations vs. bronchoscopic manifestations

General pulmonary function tests are an important method widely used to assess the nature, type, and severity of lung function impairment. Pulmonary function tests were performed on 261 children over 6 years old with MPP, including 145 in the severe group and 116 in the mild group. Through pulmonary function tests, it was found that several key pulmonary function parameters in the severe group, including total lung capacity (FVC), one-second rate (FEV1/FVC), and maximum expiratory flow (PEF), showed a significant downward trend compared with the mild group (*P* < 0.05), which was consistent with previous research results. The study found that FEF25, FEF50 and FEF75 in the lung function of SMPP children showed a downward trend, with a more significant reduction in FEF75 than in FEF25, FEF50 and MMEF, suggesting impaired small airway function in SMPP children. This result is similar to the study by Gu et al. (32), which indicated that patients with severe MPP were more prone to small airway dysfunction and decreased pulmonary ventilation function.

With the continuous advancement of medical technology, bronchoscopy has been widely promoted and applied in clinical practice. On the one hand, bronchoalveolar lavage fluid can be taken from children with MPP for mucosal biopsy, lung biopsy and cytological or etiological detection of the lavage fluid. On the other hand, through bronchoscopy, doctors can directly observe the pathological changes of the trachea and bronchi of patients. Bronchoscopy has become a safe and reliable method for diagnosing and treating children with MPP combined with pulmonary consolidation and atelectasis in clinical practice. A total of 322 children with MPP underwent bronchoscopy in this study. The proportion of those with mucus thrombus obstruction, plastic sputum thrombus, mucosal inflammatory stenosis and erosion in the severe group was significantly higher than that in the mild group, and the difference was statistically significant (*P* < 0.05), similar to the conclusion of previous studies (33). According to the latest MPP diagnosis and treatment guidelines (8), bronchoscopy should be performed as soon as possible in children with SMPP whose clinical imaging suggests atelectasis or whose mucus plug obstruction or plastic bronchitis is suspected in order to confirm the diagnosis and provide targeted treatment in a timely manner.

### 4.6 Analysis of the strengths and limitations of predictive models

The specificity of this model reached 93.5% (D-dimer cutoff value 0.91 mg/L), and the combined index sensitivity (89.5%) was also superior to that of the model based on CRP and imaging by Liu et al. (10) (sensitivity 82%); Compared with complex models that require bronchoalveolar lavage fluid testing (such as SAA, IL-6) or pulmonary function parameters (such as FEF75), the parameters of this model are more accessible and suitable for resource-limited scenarios; The AUC (0.963) of this model was significantly higher than that of Zhang et al. (34) based on NLR and PLR (AUC 0.85) and Wen et al. (29) based on D-dimer and LDH (AUC 0.89), suggesting superior disease stratification ability. However, the limitation of this study is that the validation cohort of this study was a time validation of the same medical system, and children from different regions and different levels of medical institutions were not included. The generalization of the model may be affected by factors such as regional medical level and the characteristics of the children's population. In the future, multi-center and large-sample studies will be carried out to include children with MPP in different regions and age groups to further verify and optimize the model and improve its clinical generalization ability. The diagnosis of SMPP was based on the Guidelines for the diagnosis and treatment of Community-acquired pneumonia in Children (2019 edition), in which extrapulmonary complications and the extent of pulmonary imaging involvement were the core diagnostic indicators, and these indicators were also included in the variable screening of the prediction model in this study. There were overlapping indicators, which may cause slight bias in the evaluation of the prediction efficacy of the model. Future studies will further optimize the index system for SMPP diagnosis and model construction to reduce overlapping bias.

## 5 Conclusions

The nomogram model constructed in this study, based on five indicators including ESR, albumin, LDH, lung consolidation, and D-dimer, demonstrated significant clinical predictive value. The advantages are mainly reflected in the intuitive graphical interface of the nomogram, which transforms complex mathematical models into clinically operable tools. Doctors can quickly assess the risk of SMPP based on the accumulation of patient indicator scores without relying on complex calculation formulas or professional software, especially suitable for primary care scenarios, with clinical practicality, and through multi-dimensional verification, The model was confirmed to have good calibration and clinical net benefit rate.

## Data Availability

The raw data supporting the conclusions of this article will be made available by the authors, without undue reservation.

## References

[1] Li Y Guo Z Zhang G Tian X Li Q Chen D The correlation between vitamin a status and refractory *Mycoplasma Pneumoniae* pneumonia(RMPP) incidence in children. BMC Pediatr. (2020) 20(1):359. 10.1186/s12887-020-02254-y 32731898 PMC7392651

[2] Beeton ML Zhang XS Uldum SA Bébéar C Dumke R Gullsby K *Mycoplasma pneumoniae* infections, 11 countries in Europe and Israel, 2011 to 2016. Euro Surveill. (2020) 25(2):1900112. 10.2807/1560-7917.ES.2020.25.2.1900112 31964459 PMC6976882

[3] Diksha Kamal R Narang RK Singh A. A comprehensive approach to managing respiratory illnesses among children in northern China. Infect Disord Drug Targets. (2025) 25(1):e030624230607. 10.2174/0118715265303956240514112633 38835126

[4] Yan C Xue GH Zhao HQ Feng YL Cui JH Yuan J. Current status of *Mycoplasma pneumoniae* infection in China. World J Pediatr. (2024) 20:1–4. 10.1007/s12519-023-00783-x 38185707 PMC10827902

[5] Hu SH Chen L Zhao M Ma Z Zhang H. Epidemiological characteristic analysis of *Mycoplasma pneumoniae* infection in children in Shanghai. Lab Med. (2023) 38(1):14–7. 10.3969/j.issn.1673-8640.2023.01.003

[6] Oishi T Ouchi K. Recent trends in the epidemiology, diagnosis, and treatment of macrolide-resistant *Mycoplasma pneumoniae*. J Clin Med. (2022) 11(7):1782. 10.3390/jcm11071782 35407390 PMC8999570

[7] Parums DV. Editorial: outbreaks of post-pandemic childhood pneumonia and the Re-emergence of endemic respiratory infections. Med Sci Monit. (2023) 29:e943312. 10.12659/MSM.943312 38037346 PMC10702145

[8] Zhao SY Qian SY Chen ZM. Guidelines for the diagnosis and treatment of *Mycoplasma pneumoniae* pneumonia in children (2023 edition). Infect Dis Inform. (2023) 36(4):291–7. 10.3969/j.issn.1007-8134.2023.04.002

[9] Liu SS Ma J Zhang ZX Li CX Han LL Meng C. Early predictors of *Mycoplasma pneumoniae* necrotizing pneumonia in children. Chin J Appl Clin Pediatr. (2021) 36(08):601–4. 10.3760/cma.j.cn101070-20191231-01327

[10] Liu LP Yang ZY Wang Y Shi MM Wang Y Yang YY Analysis of clinical characteristics and related risk factors of severe mycoplasma pneumoniae pneumonia in children. Chin Pediatr Emerg Med. (2023) 30(6):451–6. 10.3760/cma.j.issn.1673-4912.2023.06.010

[11] Merida-Vieyra J Aquino-Andrade A Palacios-Reyes D Murata C Ribas-Aparicio RM De Colsa Ranero A. Detection of Mycoplasma pneumoniae in Mexican children with community-acquired pneumonia: experience in a tertiary care hospital. Infect Drug Resist. (2019) 12:925–35. 10.2147/IDR.S193076 31118700 PMC6503500

[12] Experts Group of Pediatric Respiratory Endoscopy, Talent Exchange Service Center of National Health Commission Endoscopy Committee, Pediatric Section of Chinese Medical Doctor Association Pediatric Respiratory Endoscopy Committee, Endoscopists Section of Chinese Medical Doctor Association Pediatric Interventional Respirology Group, Maternal and Pediatric Minimally Invasive Section of Chinese Maternal and Child Health Association Bronchoscopy Collaboration Subgroup of Respirology Group, Pediatric Section of Chinese Medical Association Guideline of pediatric flexible bronchoscopy in China(2018 version). Chin J Appl Clin Pediatr. (2018) 33(13):983–9. 10.3760/cma.j.issn.2095-428X.2018.13.006

[13] Liu SQ Xie LY Zeng SZ Yu T. Epidemiological analysis of Mycoplasma pneumoniae infection in hospitalized children with community-acquired pneumonia in Hunan from 2013 to 2021. Chin J Microbiol Immunol. (2023) 43(06):432–41. 10.3760/cma.j.cn112309-20220914-00295

[14] Wang F Cheng Q Jing SJ Li J Zhou XD Shang YX Clinical manifestations and epidemiological characteristics of children with *Mycoplasma pneumoniae* infection in 3 hospitals of Liaoning province. Chin J Nosocomiol. (2024) 34(23):3611–7. 10.11816/cn.ni.2024-240433

[15] Gong H Sun B Chen Y Chen H. The risk factors of children acquiring refractory *Mycoplasma pneumoniae* pneumonia: a meta-analysis. Medicine (Baltimore). (2021) 100(11):e24894. 10.1097/MD.0000000000024894 33725960 PMC7982158

[16] Wang H Xu WH Liu JR Peng Y Peng XX Wen XH Clinical phenotyping of severe *Mycoplasma pneumoniae* pneumonia in children. Chin J Pediatr. (2024) 62(7):669–75. 10.3760/cma.j.cn112140-20231227-00466 38955686

[17] Ye J Liu CS. Progress of severity evaluation of *Mycoplasma pneumoniae* pneumonia in children. Int J Pediatr. (2019) 46(5):364–6. 10.3760/cma.j.issn.1673-4408.2019.05.014

[18] Yao HS Liu LY Yi LL Han LN Zhou QL Li M Clinical characteristics and risk factors for plastic bronchitis caused by severe *Mycoplasma pneumonia* in children. Chin Pediatr Emerg Med. (2021) 28(8):673–8. 10.3760/cma.j.issn.1673-4912.2021.08.007

[19] Zhang FR Zhou WF Yuqin L Luo YL Chu C. The diagnostic value of neutrophil-to-lymphocyte ratio, platelet-to-lymphocyte ratio in severe *Mycoplasma pneumoniae* pneumonia. Chin J Appl Clin Pediatr. (2022) 37(4):260–4. 10.3760/cma.j.cn101070-20201013-01612

[20] Tang Q Zhao L Zhang W Wang Z. Predictive values of neutrophil-to-lymphocyte ratio, platelet-to-lymphocyte ratio and mean platelet volume-to-lymphocyte ratio in children with refractory *Mycoplasmal pneumoniae* pneumonia. Int J Respir. (2024) 44(9):1061–6. 10.3760/cma.j.cn131368-20240428-00225

[21] Chen Y Wang J Wang XY. Analysis of influencing factors of severe *Mycoplasma pneumoniae* pneumonia in children. J Southeast Univ Med Sci Ed. (2025) 44(1):127–32. 10.3969/j.issn.1671-6264.2025.01.021

[22] Xu X Cao L Zhao AH. Construction of risk prediction model for bronchiolitis obliterans in children with refractory *Mycoplasma Pneumoniae* pneumonia based on the interpretable machine learning algorithm. Pract J Card Cerebr Pneum Vasc Dis. (2025) 33(2):20–6. 10.12114/j.issn.1008-5971.2025.00.016

[23] Pan XL Li SJ Cui WQ. Characteristics and analysis of peripheral blood WBC and serum CRP and PCT levels in children with different types of infectious pathogens. Syst Med. (2023) 8(13):150–3. 10.19368/j.cnki.2096-1782.2023.13.150

[24] Li RT Yue YC Gu XJ Xiong LL. Risk prediction models for refractory *Mycoplasma Pneumoniae* pneumonia in children: a systematic review. Chin Gen Pract. (2025) 28(9):1105–14. 10.12114/j.issn.1007-9572.2024.0098

[25] Gordon O Oster Y Michael-Gayego A Marans RS Averbuch D Engelhard D The clinical presentation of pediatric *Mycoplasma pneumoniae* infections—a single center cohort. Pediatr Infec Dis J. (2019) 38(7):698–705. 10.1097/INF.0000000000002291 30985519

[26] Liu JL. Determination of C-reactive protein and lactate dehydrogenase levels in early identification of severe *Mycoplasma pneumoniae* pneumonia. Chin Remed Clin. (2021) 21(11):1858–60. 10.11655/zgywylc2021.11.006

[27] Yuan XY Jia CM Jiang CR. Significance of hepatocyte growth factor in early diagnosis and dynamic monitoring of severe *Mycoplasma pneumoniae* pneumonia in children. J Clin Pediatr. (2021) 39(11):855–9. 10.3969/j.issn.1000-3606.2021.11.015

[28] Cheng S Lin J Zheng X Yan L Zhang Y Zeng Q Development and validation of a simple-to-use nomogram for predicting refractory *Mycoplasma pneumoniae* pneumonia in children. Pediatr Pulmonol. (2020) 55(4):968–74. 10.1002/ppul.24684 32040888

[29] Wen J Su Y Sun H Zhang H Li H. The combination of initial markers to predict refractory *Mycoplasma pneumoniae* pneumonia in Chinese children: a case control study. Respir Res. (2021) 22(1):89. 10.1186/s12931-020-01577-9 33752670 PMC7983087

[30] Nakanishi M Nakashima K Takeshita M Yagi T Nakayama T Kiguchi T Ability of high-resolution computed tomography to distinguish *Mycoplasma pneumoniae* pneumonia from other bacterial pneumonia: significance of lateral bronchial lesions, less air bronchogram, and no peripheral predominance. Respir Investig. (2020) 58(3):169–76. 10.1016/j.resinv.2020.01.006 32146120

[31] Gao H Tian JM. Logistic regression analysis of high-risk factors associated with *Mycoplasma Pneumoniae* pneumonia in paediatric patients with severe pneumonia. J Inn Mong Med Univ. (2023) 45(S1):130–3. 10.16343/j.cnki.issn.2095-512x.2023.s1.059

[32] Gu JH Jin XQ Xu YN Su YJ Cui Z Gui LQ. Analysis of changes in lung function, FeNO level and Th1/Th2 immune balance in children with mycoplasma pneumonia and wheezing. Hainan Med J. (2022) 33(5):591–4. 10.3969/j.issn.1003-6350.2022.05.013

[33] Chu FF Liang L Xu CY. Early application of soft bronchoscope in the treatment of mycoplasma pneumoniae pneumonia in children with bronchial mucus plug. J Clin Exp Med. (2023) 22(4):410–4. 10.3969/j.issn.1671-4695.2023.04.021

[34] Shen F Dong C Zhang T Yu C Jiang K Xu Y Development of a nomogram for predicting refractory *Mycoplasma pneumoniae* pneumonia in children. Front Pediatr. (2022) 10:813614. 10.3389/fped.2022.813614 35281240 PMC8916609

